# HCl Gas Sensor Coating Based on Poly(*N*-isopropylacrylamide) Nanoparticles Prepared from Water-Methanol Binary Solvent

**DOI:** 10.3390/s18103283

**Published:** 2018-09-29

**Authors:** Masanobu Matsuguchi, Shinnosuke Fujii

**Affiliations:** Department of Materials Science and Biotechnology, Graduate School of Science and Engineering, Ehime University, 3-Bunkyo-cho, Matsuyama, Ehime 790-8577, Japan; d844026m@mails.cc.ehime-u.ac.jp

**Keywords:** poly(*N*-isopropylacrylamide), cononsolvency, nanoparticle, HCl gas sensor, quartz crystal microbalance (QCM)

## Abstract

Poly(*N*-isopropylacrylamide) (PNIPAM) nanoparticles formed in water-methanol binary solvent were successfully deposited on a resonator surface at room temperature by exploiting the cononsolvency effect on the phase transition of PNIPAM aqueous solutions. Scanning electron microscopic observation revealed that the nanoparticles were secondary and made up of agglomerated primary spherical particles of about 10-nm diameter, buried in the film. The magnitude of the sensor response toward HCl gas was larger than that of the nanoparticle sensor prepared from pure water solvent, and the sensitivity to 1 ppm of HCl of sensor-coated nanoparticles based on the present method was 3.3 Hz/ppm. The recovery of the sensors was less than 90% at first cycle measurement, but had improved to almost 100% at the third cycle.

## 1. Introduction

Since HCl gas is toxic to the human body and has both direct and indirect impacts on the climate [1,2], easy and accurate monitoring of HCl gas in the environment is necessary. Various types of HCl gas sensors have been investigated, including optochemical [3,4,5,6,7,8], amperometric [9], conductometric [10,11], and solid electrochemical sensors [12]. However, each of these has drawbacks, such as limited sensitivity, response time, selectivity, or stability. As a result, research into further improvements is ongoing. Laser absorption spectroscopy (LAS) is promising, owing to its fast response, high sensitivity, and excellent selectivity compared with the chemical sensors mentioned above [13]. Quartz-enhanced photoacoustic spectroscopy (QEPAS) is another effective trace gas sensor technology. QEPAS uses a commercially available millimeter-sized piezoelectric quartz tuning fork as an acoustic wave transducer [14]. However, these sensors also have disadvantages, such as high operation temperature, bulky size, and high cost.

The object of our current work is to design a highly sensitive, but simple, small, and low-cost HCl gas sensor that is operable near room temperature. Toward this end, we have chosen a quartz crystal microbalance-based sensor coated with various sensing polymers that have basic groups as acidic HCl gas adsorption sites. However, with this approach it is often necessary to make a choice between the realization of high adsorption ability and that of reversible adsorption. That is, the introduction of a strong adsorption site in the chemical structure of a polymer enhances adsorption ability but also makes adsorption irreversible. This dilemma has made the development of practical sensor coatings difficult.

We have been studying the application of poly(*N*-isopropylacrylamide) (hereafter, PNIPAM) to a mass-sensitive HCl gas sensor coating [15]. PNIPAM is the most popular and well-investigated thermo-responsive polymer, and it has a lower critical solution temperature (LCST) of around 32 °C. Since PNIPAM chains shrink to take a globular form above LCST [16,17,18], this characteristic can be utilized to prepare nanoparticles [19,20]. On the other hand, thin films also can be prepared by coating PNIPAM solution at below LCST. We have shown that differences in the morphology of coated PNIPAM significantly affected sensing characteristics [21]. Specifically, the thin film had a greater sensor response, but the response time was long and reversibility was poor. On the other hand, the nanoparticle form showed a smaller sensor response but high reversibility, irrespective of PNIPAM loading on the quartz resonator. Moreover, the response of the nanoparticle sensor could be enhanced by increasing the mass of loading without losing reversibility. These results suggest that nanoparticle sensors have the potential to solve the dilemma outlined above, and can be used for the continuous monitoring of HCl gas concentrations at room temperature.

However, despite these promising results, the applicability of nanoparticle sensors was limited by a problem in the production of PNIPAM nanoparticle coatings. Initially, PNIPAM nanoparticles were prepared by precipitation polymerization in a pure water solvent via atom transfer radical polymerization (ATRP) at 45 °C [20]. The resultant monodispersed solution of nanoparticles was then drop-coated onto the resonator surface through a series of processes, including centrifugal separation, filtration, re-dispersion in water, coating, and drying. However, because the nanoparticles are soluble in water below LCST, it was necessary to perform these complicated deposition processes at high temperature (80 °C). The formation of densely packed PNIPAM nanoparticles was difficult under these conditions, and the production of sensor devices often lacked reproducibility. Consequently, this fabrication technique required a lot of labor and time, and was inadequate for the mass production of sensing elements.

To overcome this limitation, we proposed a new fabrication process for PNIPAM nanoparticles, which we called the “spray-coating method”. This procedure consisted of simply spraying PNIPAM dissolved in water below LCST onto a heated quartz resonator surface at 50 °C [22]. The spraying induced the formation of aggregates on the resonator surface, presumably due to a coil-to-globule transition. Thus, nanoparticles were successfully formed through simple processes; however, the reversibility of HCl gas adsorption of the resultant coating was insufficient compared with that of nanoparticles prepared by the former method. This insufficient reversibility was attributed to the formation of loosely cross-linked nanoparticles in which the internal hydrogen bonding was not well developed. The low density of nanoparticles in nature allowed the adsorption of HCl gas inside the nanoparticles, resulting in insufficient reversibility.

In this work, we propose a newly developed method for fabricating PNIPAM nanoparticle sensors in which all processes can be performed at room temperature. To accomplish this, we utilized a well-known cononsolvency effect on the phase transition of PNIPAM aqueous solutions. Cononsolvency is a phenomenon in which the addition of good organic solvents to an aqueous PNIPAM solution promotes a drastic change in the LCST [23,24,25,26,27,28]. In particular, the addition of small amounts of solvents such as methanol decreases the transition temperature. In this study, PNIPAM nanoparticles were prepared in a water-methanol binary solvent with methanol mole fractions of 0.2 and 0.3, since the reported LCSTs of PNIPAM in these solutions are below the laboratory temperature [23,24,28]. The resultant nanoparticle dispersion solutions in binary solvents were coated onto a quartz resonator at room temperature. Then, the HCl gas adsorption/desorption properties of the nanoparticles were investigated with a quartz crystal microbalance (QCM) technique for the HCl gas sensor coating application.

## 2. Materials and Methods

### 2.1. Preparation of the PNIPAM Nanoparticle Sensor

The PNIPAM nanoparticle sensor was prepared as follows. All chemicals were analytically pure, and used without further treatment.

A PNIPAM homopolymer was synthesized at 25 °C in water via ATRP [29]. First, 0.1 mmol of Cu(I)Br (Wako Pure Chemicals) was dissolved as a catalyst in 20 mL of pure water, which was degassed in advance by bubbling N_2_ to remove dissolved oxygen. Then 0.1 mmol of Tris [2-(dimethylamino)ethyl]amine (Me_6_TREN) (Sigma-Aldrich, Tokyo, Japan) was added to the flask as a ligand. Next, 5.0 mmol of NIPAM monomer (Wako Pure Chemicals) in 10 mL of degassed water was added to the flask, followed by 0.1 mmol of 2-bromoisobutyryl bromide (BIBB) (Tokyo Chemical Industry Co. Ltd., Tokyo, Japan) as a polymerization initiator in 10 mL of degassed water. The solution was stirred for 24 h to allow polymerization while bubbling with N_2_ gas. Polymerization was terminated by adding 1 mL of 1 M HCl, and then the solution was dialyzed with distilled water using a dialysis membrane (molecular weight cutoff = 3500).

The dispersion solution of PNIPAM nanoparticles in water-methanol binary solvent was prepared as follows. First, methanol was added to the purified PNIPAM aqueous solution described above at the desired concentration with a methanol mole fraction (*X*_M_) of 0.2 or 0.3. The produced PNIPAM nanoparticles were then subjected to centrifugal separation. Finally, the separated nanoparticles were dispersed into the binary solvent (*X*_M_ = 0.2 and 0.3) again.

Each dispersion solution of PNIPAM nanoparticles was drop-coated onto one side of an AT-cut quartz resonator (9 MHz) surface with a gold electrode at room temperature and dried under reduced pressure. The mass of the loading was controlled by changing the amount of solution dropped.

### 2.2. Measurement of Sensing Properties

All sensors were set in a thermostated measuring chamber. The experimental setup was reported previously [21]. Measurement was performed at 30 °C in flows of 96 ppm of dry HCl gas diluted with N_2_. Exposure and removal of HCl gas were repeated for three cycles. The variation in the resonance frequency of the quartz resonator was measured with a quartz crystal microbalance system (QCA 922; Seiko EG&G Co., Ltd., Tokyo, Japan,). Damping measurement was performed together with the frequency measurement throughout this study and the negligible viscoelastic contribution to the frequency shift was confirmed.

### 2.3. LCST Measurement

The lower critical solution temperature (LCST) of the PNIPAM in the water-methanol mixed solvent was determined as follows. PNIPAM solutions were cooled in an ice bath, and the resulting clear solution was transferred to a quartz cell with a 1 cm light-path length. The transmittance of the solution at 500 nm of the solution was recorded at progressively increasing temperature on a UV/Vis spectrophotometer (V-530; JASCO, Tokyo, Japan).

### 2.4. DLS Measurement

The size distribution profile of PNIPAM nanoparticles in the water-methanol mixed solvent was determined at room temperature by a dynamic light scattering (DLS) technique (Malvern; Zetasizer Nono-S). All samples were diluted appropriately with the binary solvent for the measurement, and then transferred to the quartz cell with a 1-cm light-path. Measurements were performed three times for each sample to obtain an average diameter.

### 2.5. SEM Observation

The morphologies of the PNIPAM nanoparticles coated on the resonator surface were observed using a scanning electron microscope (SEM) (Miniscope TM-1000; Hitachi High-Technologies, Shenzhen, China) and a field emission scanning electron microscope (FE-SEM) (S-5500; Hitachi High-Technologies).

## 3. Results

### 3.1. Properties of PNIPAM Solution

**Figure 1:**
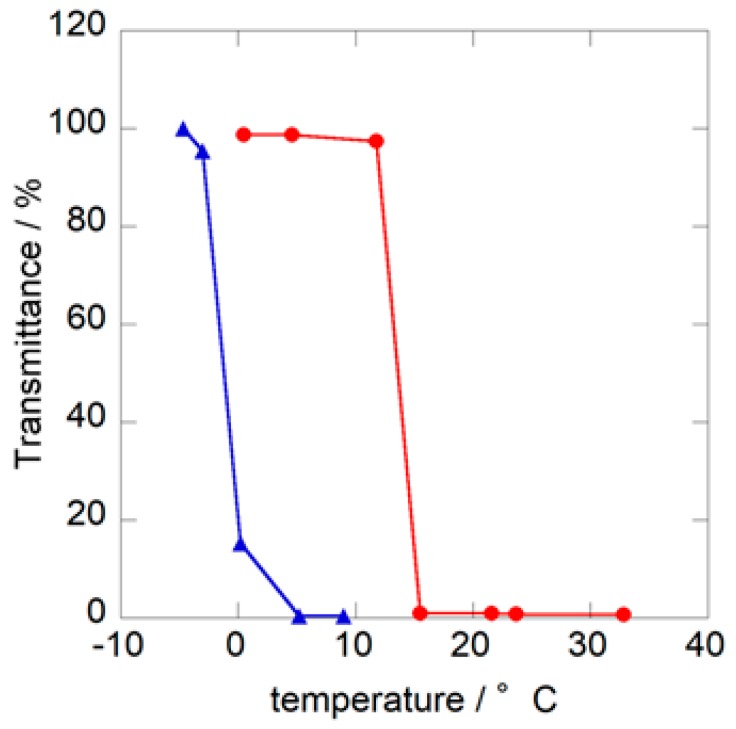
Temperature dependence of transmittance for the PNIPAM solution; (●) *X*_M_ = 0.2; (▲) *X*_M_ = 0.3.

**Figure 2:**
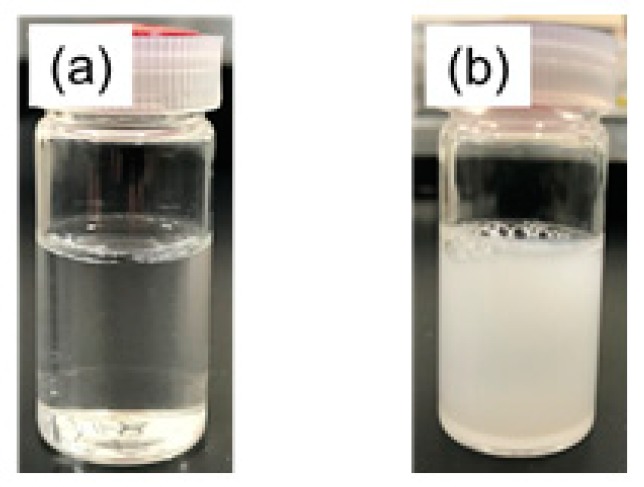
PNIPAM solutions at 25 °C: (**a**) *X*_M_ = 0; (**b**) *X*_M_ = 0.20.

“Cononsolvency” is a phenomenon in which the addition of good organic solvents to aqueous PNIPAM solution promotes a drastic change in the LCST. The origin of this phenomenon was theoretically explained by Tanaka et al. [30]. The LCSTs of PNIPAM in water-methanol solvent mixtures containing an intermediate amount of methanol are known to be below the laboratory temperature [23,24,28]. Figure 1 shows the change in transmittance of the PNIPAM solutions with *X*_M_ = 0.2 and 0.3 as a function of temperature. The addition of methanol promotes a decrease in LCST from 32 °C in pure water to below room temperature. Moreover, LCST clearly depended on the mole fraction of methanol in binary solvent, and the estimated LCSTs were 12 °C for the *X*_M_ = 0.2 solution and −3 °C for the *X*_M_ = 0.3 solution. These results are in good agreement with those reported by Freitas et al. [24]. Indeed, as shown in Figure 2, the PNIPAM solution in pure water (*X*_M_ = 0) was clear at 25 °C, but that of the *X*_M_ = 0.2 solution was milky, indicating that PNIPAM particles were formed in water-methanol mixed solvent even at 25 °C.

**Figure 3:**
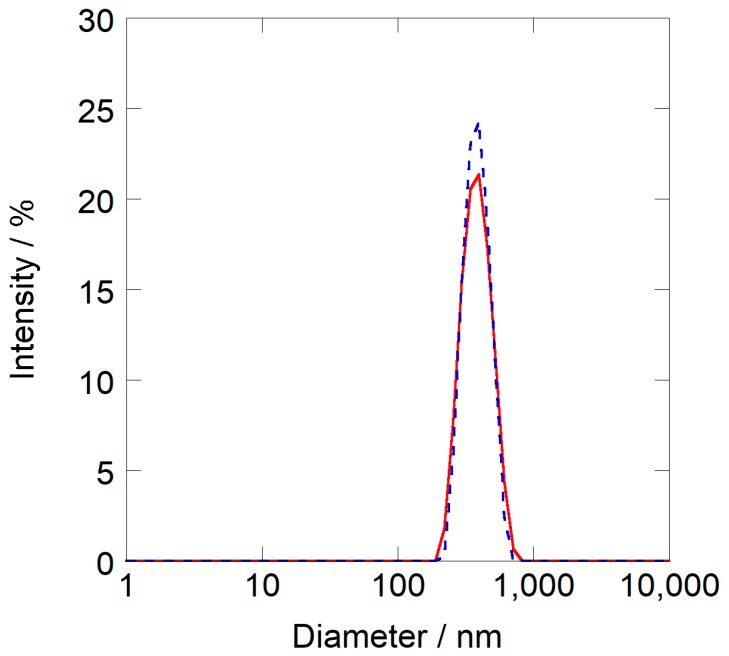
Size distribution of PNIPAM nanoparticles (measured by dynamic light scattering study) in water-methanol binary solvent: red line (*X*_M_ = 0.20); black dotted line (*X*_M_ = 0.3).

Figure 3 shows the average diameter of the nanoparticles formed in each binary solvent measured by dynamic light scattering (DLS). The DLS analysis demonstrated that the nanoparticles were monodistributed in the water-methanol mixed solvent. Moreover, the average diameters of the nanoparticles in these solutions were independent of the compositions of binary solvent, and were around 340–380 nm.

### 3.2. Morphology of PNIPAM Nanoparticle Coating

**Figure 4:**
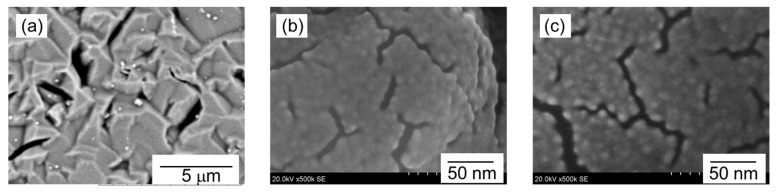
SEM images of the resonator surface coated with PNIPAM nanoparticles; (**a**) low magnification (×10 k); (**b**) high magnification (×500 k) of the nanoparticle observed in (**a**); (**c**) high magnification (×500 k) in the region where nanoparticles were not observed in (**a**).

SEM images of the resonator surface coated with PNIPAM nanoparticles formed in a solution of *X*_M_ = 0.2 are shown in Figure 4. The image in Figure 4a was obtained at low magnification (×10 k). It should be noted that the observed roughness of the surface was attributed to the irregularity of the gold electrode on the resonator surface. As can be seen, nanoparticles ranging from 100 to 300 nm were sparsely distributed on the rough resonator surface. The average nanoparticle size was equivalent to that obtained by the DLS analysis (Figure 3). We could therefore confirm that PNIPAM nanoparticles formed in the water-methanol solution were successfully deposited on the resonator surface at room temperature.

On the other hand, as shown in Figure 4b, SEM observation at high magnification revealed that the nanoparticles observed in Figure 4a are secondary, and consist of agglomerated primary spherical particles of about 10 nm in diameter. Moreover, as shown in Figure 4c, primary particles can also be observed in the region where nanoparticles were not observed at low magnification. These primary particles appear to be buried in the film; this morphology was also observed when nanoparticles were coated from the solution of *X*_M_ = 0.3. The formation mechanism of the primary particles is unknown at the present stage.

### 3.3. Response Toward HCl Gas

**Figure 5:**
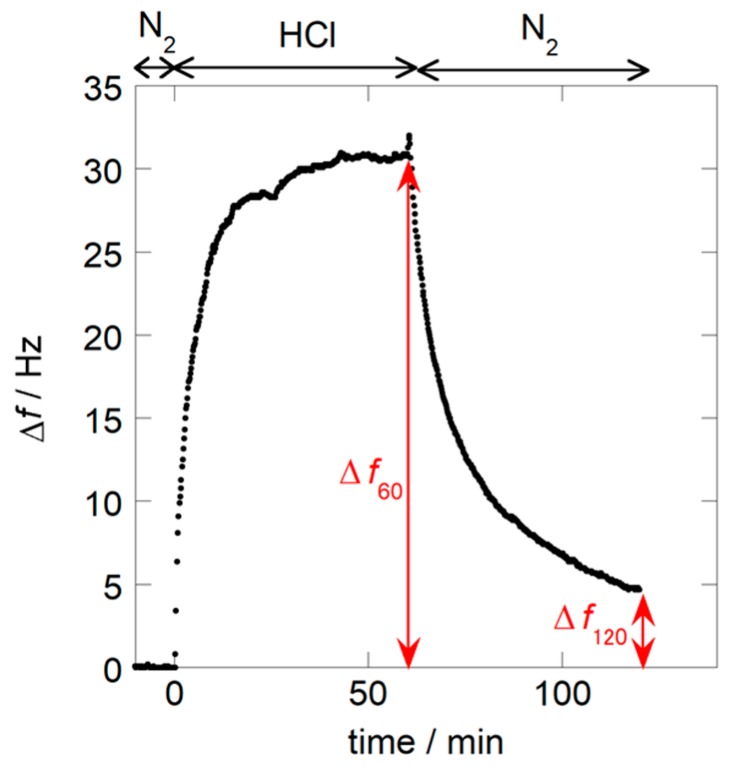
Typical response of the PNIPAM nanoparticle sensor to 98 ppm of HCl/N_2_ at 30 °C.

A typical response of the present nanoparticle sensor obtained at the first cycle measurement is shown in Figure 5. HCl adsorption on the nanoparticle coating leads to a decrease in resonant frequency, and results in a positive Δ*f*. This figure also shows the recovery of the response. After the sensor was exposed to dry HCl gas for 60 min, the dry N_2_ atmosphere was restored for 60 min. The frequency shift then reverted toward the initial value and reached Δ*f*_120_. Here, we defined two sensing characteristics. The first is the magnitude of the response Δ*f*_60_, defined as the frequency shift after 60 min of exposure to HCl gas. This value is equivalent to the HCl gas adsorption capacity of the nanoparticle coating. The second is the recovery, *Re*, which describes the recovered value after 60-min re-exposure to N_2_ gas. This value is expressed as a percentage of the initial magnitude of response, and calculated according to Equation (1).

(1) $$Re\left(\%\right)=\frac{\Delta f_{60}-\Delta f_{120}}{\Delta f_{60}}\times 100\;$$

This value shows the reversibility of HCl adsorption/desorption of the nanoparticle coating.

**Figure 6:**
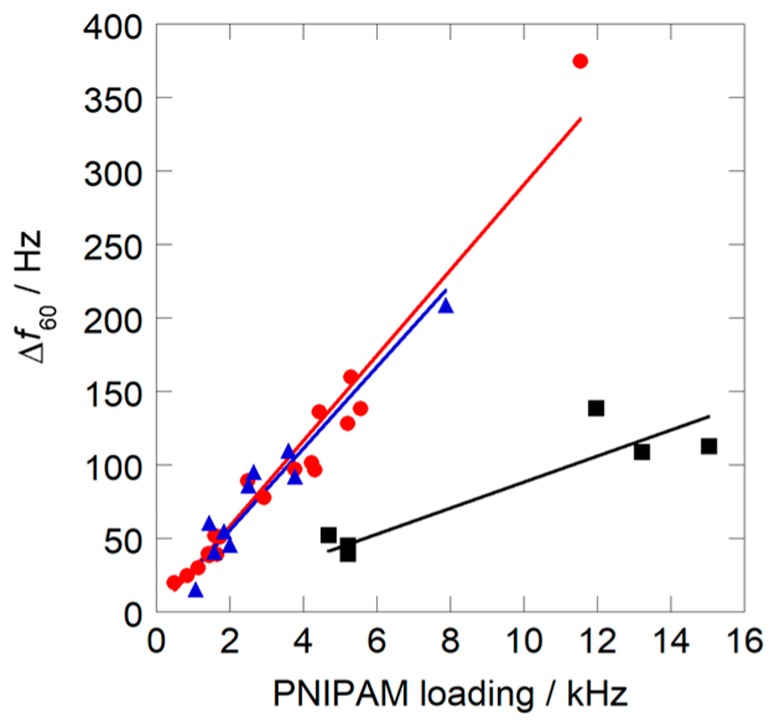
Dependence of the magnitude of the sensor response on PNIPAM loadings measured at 30 °C: (●) *X*_M_ = 0.2; (▲) *X*_M_ = 0.3; (■) *X*_M_ = 0.

**Figure 7:**
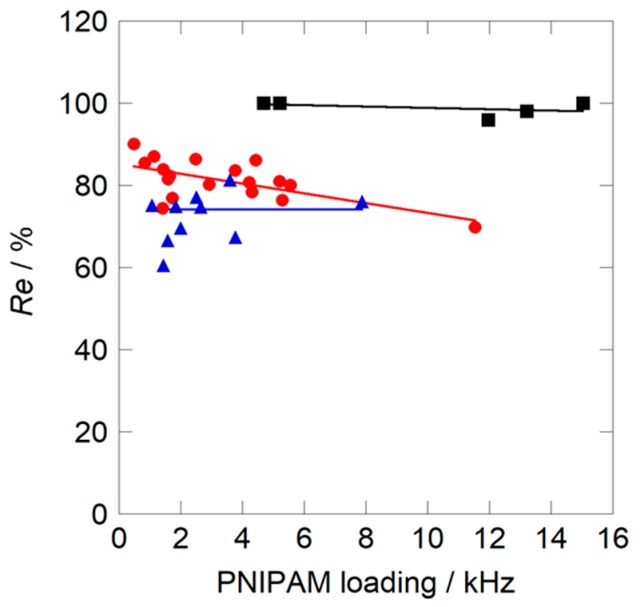
Dependence of the recovery of the sensor on PNIPAM loadings measured at 30 °C: (●) *X*_M_ = 0.2; (▲) *X*_M_ = 0.3; (■) *X*_M_ = 0.

Figure 6 shows the relationships between the magnitude of the response Δ*f*_60_ and PNIPAM loadings on the resonator surface coated by solutions with different compositions obtained at the first cycle measurement. For all sensors, Δ*f*_60_ values increased linearly as PNIPAM loading increased. Among the sensors, those coated with nanoparticles prepared in water-methanol binary solvent showed larger slopes, irrespective of their composition, compared to those coated with pure water solvent prepared by the former method. This result indicated that the coatings from water-methanol binary solvent had larger HCl gas adsorption capacities. On the other hand, while the recovery of the nanoparticle sensor coated from pure water solvent was 100% irrespective of PNIPAM loading, the recovery of the sensors from binary solvents declined to less than 90%, as shown in Figure 7.

**Figure 8:**
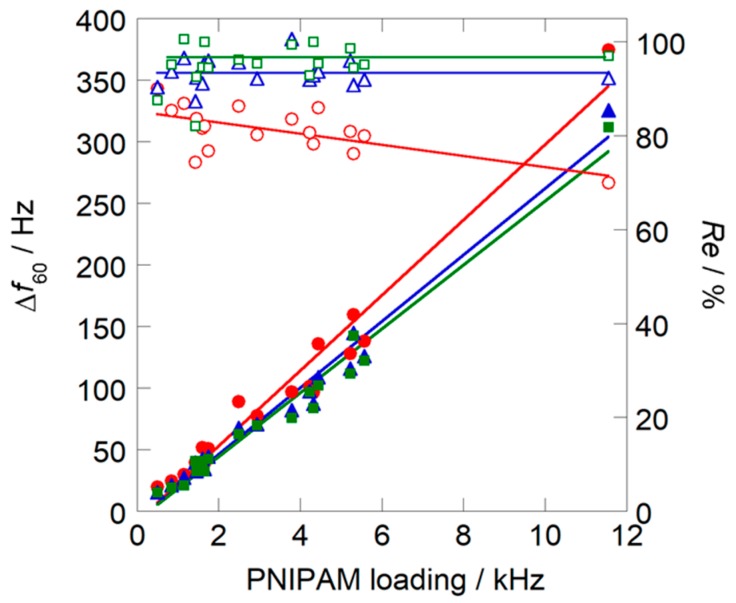
Dependence of the magnitude of the sensor response and recovery on PNIPAM loadings measured at 30 °C: (●) first cycle; (▲) second cycle; (■) third cycle. Closed symbols show the sensor response; open symbols indicate the recovery.

In this study, response measurement was repeated for three successive cycles. Figure 8 shows that recovery of the sensors from the binary solvent improved with increasing number of measurement cycles, and reached close to 100% at the third cycle of measurement. Moreover, the magnitude of the response was scarcely decreased by the increase in measurement cycles, and was still three times larger than that of the pure water solvent sensor. The sensitivity to 1 ppm of HCl could be calculated from this frequency shift, and was 3.3 Hz/ppm when PNIPAM loading was 11.6 kHz. These results suggest that the present nanoparticle sensor prepared from binary solvent could be applied to continuous monitoring of HCl concentrations in work places at room temperature.

## 4. Discussion

**Figure 9:**
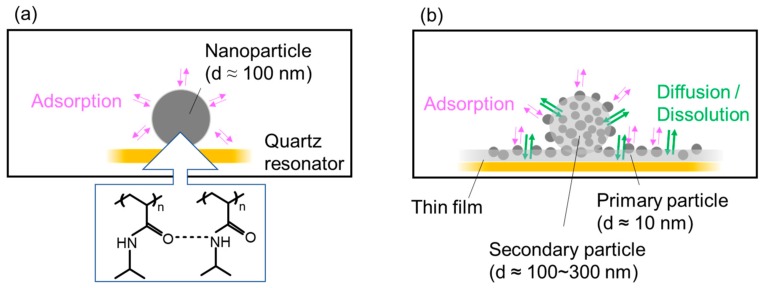
HCl gas adsorption/desorption mechanism: (**a**) nanoparticle coating from pure water solvent; (**b**) nanoparticle coating from water-methanol binary solvent.

We will consider why the solvent of the PNIPAM nanoparticle solution affected the HCl gas adsorption characteristics of the resulting nanoparticle coating. For these purposes, we will assume that the HCl adsorption site was the C=O group on the PNIPAM chain. Then, the possible adsorption mechanisms on those nanoparticle coatings can be schematically illustrated as shown in Figure 9. In our previous study, we described the complete reversibility of HCl gas adsorption/desorption of a nanoparticle coating prepared from pure water solvent, as shown in Figure 9a [22]. The nanoparticles from the pure water solvent had a mean diameter of approximately 100 nm. Moreover, FT-IR analysis confirmed that the nanoparticles had high density, due to the highly developed hydrogen bonding between the isopropyl groups they contained. Consequently, the adsorption seemed to occur only in the vicinity of the surface of the nanoparticles, leading to completely reversible adsorption/desorption, but also to a small sensor response.

On the other hand, as shown in Figure 9b, SEM observation in the present study revealed that the nanoparticles prepared from water-methanol binary solvent were secondary and consisted of agglomerated primary particles (d ≈ 10 nm) that were buried in the formed film. When the film form co-exists with the primary nanoparticles, gas adsorption occurs on the primary particle surface and film surface, followed by dissolution and diffusion into the film. As a result, the coating shows high gas adsorption capacity due to both the large specific surface area derived from small primary particles and the adsorption on non-hydrogen bonding C=O groups inside the film. However, the diffusion process is kinetically slow, which leads to a slow response rate and poor reversibility. This may be attributable to a specific characteristic of the present nanoparticle coating—namely, its incomplete recovery from the binary solvent observed at the first cycle. However, this slow diffusion process can be ignored in continuous measurement. Consequently, the response decreased but the reversibility improved with the measurement cycle, and *Re* values were approximately 100%, irrespective of the PNIPAM loading at the third cycle.

## 5. Conclusions

PNIPAM nanoparticle coating is a promising HCl gas sensing material that has the potential to resolve the trade-off between the realization of high adsorption ability and the realization of reversible adsorption. In this study, by exploiting the cononsolvency effect on the phase transition of PNIPAM aqueous solutions, we successfully coated PNIPAM nanoparticles onto the surface of a quartz crystal resonator at room temperature. This would significantly reduce the labor and time required to mass-produce nanoparticle sensors for HCl gas detection. The magnitude of the sensor response toward HCl gas was better than that of the nanoparticle sensor prepared from pure water solvent. Recovery was less than 90% at the first cycle measurement due to the simultaneous formation of the primary particles and the thin film; however, this poor reversibility seemed to be irrelevant to the problem of continuous monitoring after the conditioning the coating. These results suggest that the present nanoparticle sensor prepared from a binary solvent has great potential for application to continuous monitoring of HCl concentrations in work places at room temperature.

The objective of this work is to conduct basic studies into development of mass-sensitive HCl gas sensor coatings. Fundamental findings like those reported herein will lead to a new approach to the design and development of novel gas-sensor coatings. In the future, we will attempt to investigate other sensing characteristics for practical use, such as selectivity and long-term stability.
